# Experimental and Numerical Investigation of Mechanical Properties of Hyper Polylactic Acid (HPLA)

**DOI:** 10.3390/polym18050624

**Published:** 2026-03-03

**Authors:** Mariana Domnica Stanciu, Horațiu Drăghicescu Teodorescu, Ionuț Teșulă, Sergiu Valeriu Georgescu, Florin Dinulică

**Affiliations:** 1 Department of Mechanical Engineering, Transilvania University of Brașov, B-dul Eroilor 29, 500036 Brașov, Romania; 2 Schaeffler România, Aleea Schaeffler 3, 507055 Cristian, Brașov, Romania; 3 Department of Wood Processing and Design of Wood Products, Transilvania University of Brașov, B-dul Eroilor 29, 500036 Brașov, Romania; 4 Faculty of Silviculture and Forest Engineering, Transilvania University of Brașov, B-dul Eroilor 29, 500036 Brașov, Romania

**Keywords:** hyper polylactic acid, raster angle orientation, mechanical properties, finite element analysis

## Abstract

Polylactic acid (PLA) is one of the most widely used materials for fused filament fabrication (FFF) or fused deposition modeling (FDM), being recognized for its low carbon footprint, relatively low costs and good mechanical properties. Improving the mechanical and technological properties of PLA with various additives has led to the production of different types of PLA-based filaments, such as hyper PLA (HPLA), PLA, PLA+ and PLA Lite. Studies on the mechanical properties of HPLA are scarce; therefore, the objective of this paper was to determine the mechanical properties of 3D-printed HPLA under tensile and bending stress conditions and to obtain numerical models that depend on the raster pattern orientation. The principal component analysis (PCA) reveals very different results for bending compared with tension, with outcomes varying significantly depending on the orientation of the raster angle.

## 1. Introduction

Digital manufacturing is a new technology that has, in recent years, changed the way products are designed (computer-aided design, CAD), manufactured (computer-aided manufacturing, CAM) and virtually tested (computer-aided engineering, CAE) using computers, as shown by studies [1,2,3,4]. The advantage of additive manufacturing (AM) is that it creates the geometry of the part during production by adding successive layers of material in which the upper layers merge with the previous ones, eliminating technological waste and enabling the manufacture of complex shapes with high execution precision. One of the most widespread AM methods is based on filament extrusion technology, where the feed material is in the form of a thin filament spool that is fed into a heating chamber [4,5,6]. The filaments used in fused filament fabrication (FFF) or fused deposition modeling (FDM) processes are made of different polymer materials: polylactic acid (PLA), acrylonitrile butadiene styrene (ABS), carbon fiber filament (CFF), nylon (also known as polyamide (PA)), flexible filaments (FLEX) based on a flexible copolymer from the thermoplastic polyurethane family, high-impact polystyrene (HIPS), polyvinyl alcohol filament (PVA), polyethylene terephthalate glycol-modified (PETG), thermoplastic elastomers filament (TPE), and polycarbonate filament (PC) [7]. The main characteristics of these types of filaments, according to [7], are summarized in Supplementary Materials—Table S1. Among the process parameters, such as bead width, air gap, pattern construction temperature, raster orientation, infill pattern and color, those that influence the mechanical properties of printed samples are mainly deposition line width and printing orientation, as highlighted by [8,9,10,11,12,13]. Among the different infill patterns analyzed by [13,14,15], the gyroid and honeycomb types present the most suitable ratio of material consumption, low density and high mechanical properties [16,17]. Liu et al. [18] statistically analyzed the influence of five factors in the printing process, namely deposition orientation, raster gap, raster width, deposition style, and layer thickness, at different loads and found that the most important influencing factor is deposition orientation. For PLA samples, refs. [17,18,19,20,21,22] analyzed the impact of process conditions and filament orientation angles on the tensile properties of 3D-printed PLA specimens. Besides PLA filaments, which are biodegradable and bioactive thermoplastic aliphatic polyesters with the chemical formula (C_3_H_4_O_2_)_n_, the addition of different natural fillers (biological or mineral) aims to improve the elastic properties and density, as is the case for hemp and hemp inflorescence inserts, eggshell or coconut shell powders studied by [23,24], charcoal particles analyzed by [25], and other materials, such as metal or ceramic powders as highlighted in [26]. The effect of adding fillers, such as glass fibers, carbon fibers or bronze particles, in the PLA composition on the tensile and flexural elastic properties was studied by [27]. The studies conducted by [28,29] show that technologies and research on polymer composite materials have led to the production of new types of PLA that contain, in addition to the basic matrix, various additives, such as coupling agents (mainly silanes and polymers grafted with maleic anhydride), impact modifiers (such as elastomeric polymers or other hardening agents), processing aids (such as lubricants and plasticizers), nucleating agents and antioxidants (such as talcum powder, antioxidants and UV absorbers), flame retardants and colorants. Thus, the most well-known types of PLA-based filaments on the market are hyper PLA, PLA, PLA+ and PLA Lite.

There is limited classification of “hyper PLA” within the literature and a lack of a clear explanation of how hyper PLA differs from conventional PLA. According to the manufacturer, hyper PLA contains a modified formulation of PLA, which is a biodegradable thermoplastic aliphatic polyester derived from renewable resources like cornstarch. It has a high flow, is designed for high-speed 3D printing (up to 600 mm/s), and is characterized by increased fluidity and fast curing time. It is composed of polylactic resin (approximately 98%) and calcium carbonate (CaCO_3_, approximately 2%) to improve rigidity and printing characteristics. Although considered non-toxic and with low odor, when heated, HPLA releases volatile organic compounds (VOCs) and ultrafine particles (UFPs). Data on the mechanical performance of this PLA variant is not yet reported in the state of the art.

The purpose of this study is to analyze the elastic tensile and flexural properties of samples 3D printed with hyper PLA filament—denoted HPLA—in four raster angle orientations through experimental tests and numerical simulation. The novelty of the study lies not only in the studied filament, as there has been little research to date in this field, but also in the numerical simulation of the tensile and bending behavior of HPLA with different raster orientation angles.

## 2. Materials and Methods

### 2.1. Sample Preparation

**Figure 1:**
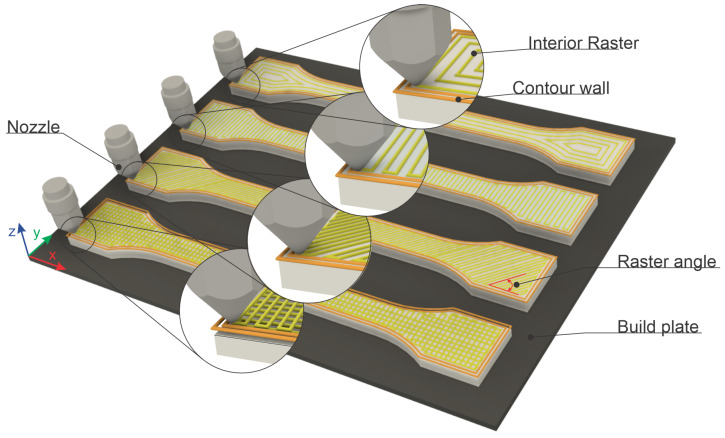
FDM toolpath parameters for obtaining sample types.

Considering that the mechanical behavior of FDM-printed samples depends on the build orientation, bead width, layer thickness, filament raster angle, raster width, and air gap [29,30,31], in this study, the raster angle was analyzed as a parameter, with the filament being made of hyper PLA. The mechanical characteristics of samples made from HPLA filament, according to the hyper PLA Filament Technical Data Sheet from Creality, are presented in Supplementary Materials—Table S2. A new/sealed roll of Creality hyper PLA filament was used for the manufacture of the specimens. As the PLA is not strongly hygroscopic, additional drying of the filament was not necessary. The environmental conditions were in accordance with the ISO 527 [32] and GB/T 1040 [33] standards specified in the product data sheet. In Figure 1, the printing process of the samples is highlighted. In the literature [34,35,36,37], the raster angle is also associated with raster orientation, layer orientation, fiber orientation, or even pattern orientation. It is defined as the angle of the filament direction with respect to the x-axis of the build plate (Figure 1). Thus, taking into account the fact that the raster direction depends on the orientation of the extruder head when the printing material is filled into the contour of the parts, the analyzed samples were printed as follows: with a raster direction angle of 45° relative to the longitudinal axis, marked HPLA45; with a raster direction angle of 90° relative to the longitudinal axis, marked HPLA90; with a crossed raster direction of +0/90°, marked HPLA0/90; and with a concentric raster pattern, marked HPLA0, all having an infill density of 100%. Regarding build orientation, the flat printing orientation was applied to all types of samples [30,34]. Two contour walls were deposited along the edge of the samples, as can be seen in Figure 1.

The printing of the samples was done using the Creality K1Max 3D printer (Shenzhen Creality 3D Technology Co., Ltd., Shenzhen, China) by fused deposition modeling (FDM). The main printing parameters used to obtain the samples are presented in Table 1. Sequences from the printing program with highlighting of the printing parameter settings are presented in Supplementary Materials—Table S3.

### 2.2. Experimental Setup

#### 2.2.1. Tensile Test

**Figure 2:**
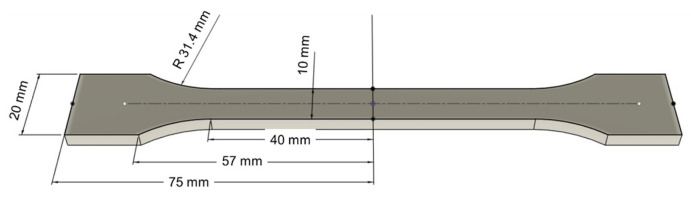
The dog-bone samples for the tensile test.

To determine the elastic characteristics of HPLA samples with different fused deposition modeling orientations, the samples were subjected to a static tensile test. In this study, the specimens were tested on the universal testing machine LS100 Lloyd’s Instrument (AMETEK Test & Calibration Instruments, Largo, FL, USA), with a 100 kN load cell. The specimens with physical features presented in Table 2 were loaded with a constant speed of 5 mm/min until breaking. The strains in the tensile direction were measured by means of an extensometer with a gauge length of 50 mm. For data acquisition, Nexygen Plus software (version 1.1.0.2) was used. The tensile tests with geometry and dimensions samples presented in Figure 2 were performed according to SR EN ISO 527-2:2025 [32]. The stress–strain curve, specific deformation, longitudinal elastic modulus, and rupture tensile strength of each tested sample were determined, and based on the load curves, the average deformation energy for each type of sample was calculated [38,39,40]. The fracture of the samples was analyzed with optical devices.

**Figure 3:**
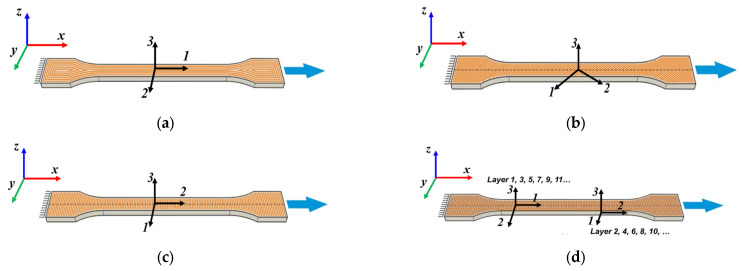
The types of samples for tensile tests with the principal loading axes (*x-y-z*) and principal materials axes (1-2-3): (**a**) tensile sample with concentric pattern; (**b**) tensile sample with 45° raster angles; (**c**) tensile sample with 90° raster angle; (**d**) tensile sample with 0/90° raster angles.

From a mechanical point of view, for each category of samples, the global coordinate system *x*, *y*, *z* was established, with the *x*-axis corresponding to the longitudinal direction of the samples and the loading direction. Depending on the raster angle orientation, a local coordinate system was established, denoted by 1, 2, and 3, as can be seen in Figure 3.

#### 2.2.2. Flexural Test

**Figure 4:**
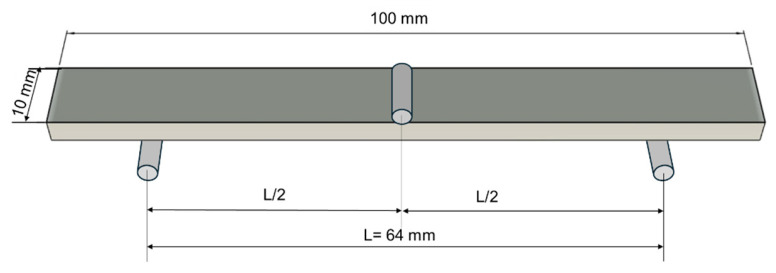
The principles of the three-point bending test.

**Figure 5:**
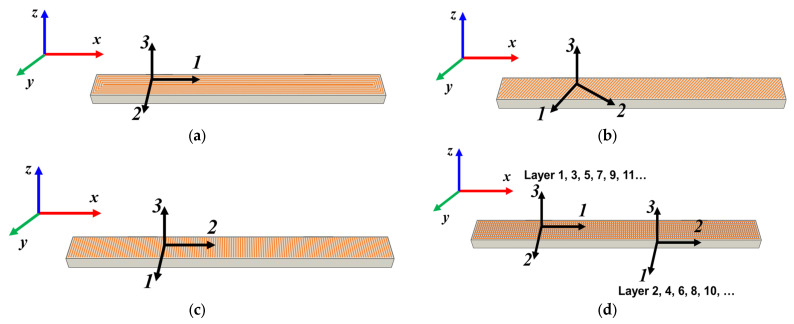
The types of samples for flexural tests with the principal loading axes (*x-y-z*) and principal materials axes (1-2-3): (**a**) flexural sample with concentric pattern; (**b**) flexural sample with 45° raster angles; (**c**) flexural sample with 90° raster angles; (**d**) flexural sample with 0/90° raster angles.

The flexural test, or the three-point bending test, was also performed in accordance with standard ISO 178:2019 [41] using the same universal testing machine presented in Section 2.2.1 but with bending devices. The span between supports was 64 mm, and the bending load was applied at 5 mm/min (Figure 4). The characteristics of the bending samples are presented in Table 3. The stiffness, flexural modulus, load, stress and deflection were determined. In Figure 5, the bending tests are presented with the global coordinate system. The force was applied in the *z* direction, producing a bending moment along the y-axis, with the local coordinate system indicating the raster angle direction.

#### 2.2.3. Statistical Analysis

Statistical data processing was performed with STATISTICA 8.0 (StatSoft 2007) [42]. The experimental data processing strategy consisted of the following stages: data exploration (initial analysis of data variability); correlation analysis based on exploring relationships between variables; dimensionality reduction based on principal component analysis (PCA), which led to the identification of the mechanical variables that are most affected by the raster angle; validation and classification (discriminant analysis) by confirming the hierarchy of these influential variables; and finally, stratification of the validated data based on the raster angle. Among the experimentally determined mechanical parameters, the following quantities were considered as initial variables:

- From the tensile test (T): stiffness (N/m) (denoted T_Stiffness); Young’s modulus (MPa) (denoted T_MOE); load at maximum load (kN) (T_Load); stress at maximum load (MPa) (T_Stress); extension at maximum load (mm) (T_Extension); percentage strain at maximum extension (%) (T_Strain); load at break (kN) (T_L Break); stress at break (MPa) (T_S Break); percentage strain at break (%) (T_Strain Brake); and tensile strength (MPa) (T_Strength).
- From the bending test (B): stiffness (N/m) (denoted B_Stiffness); Young’s modulus (MPa) (denoted B_MOE); load at maximum load (kN) (B_Load); stress at maximum load (MPa) (B_Stress); extension at maximum load (mm) (B_Extension); flexural rigidity (Nm2) (B_Rigidity); maximum bending strain at maximum extension (%) (B_Strain); work to maximum extension (Nmm) (B_Work); extension at maximum load (mm) (B_Extension).

### 2.3. Numerical Simulation

**Figure 6:**
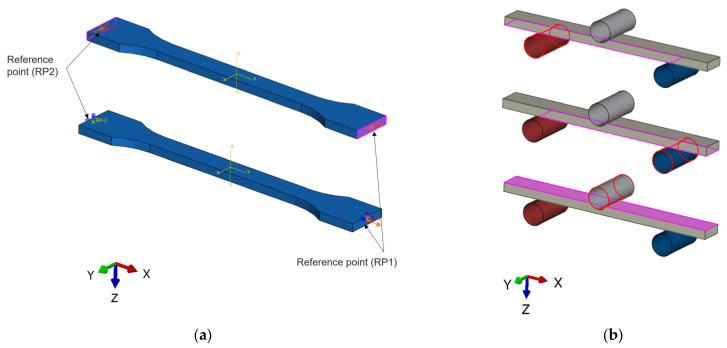
Boundary conditions and the applied loading to tensile (**a**) the contact between the bending specimen and the supports (**b**).

The numerical simulation was performed using Abaqus/CAE 2023 HF6 (version 6.0) for the pre-processing stage, Abaqus/standard 2023 HF6 for the solver, and Abaqus/CAE 2023 HF6 for the post-processing stage. The CAD model was meshed in hexahedral finite elements of type C3D81, with 8-node linear brick. For each sample, a local coordinate system was established that considered the raster angle orientation, as can be seen in Supplementary Materials—Figure S1. In the case of traction simulation, the boundary conditions were established by means of reference points created at the ends of the samples. The RPs relate to a kinematic coupling to the end surface. At reference point 1 (RP1), the load was applied, and at reference point 2 (RP2), all degrees of freedom (DOF) were fixed, as can be seen in Figure 6a. In the case of the 3-point bending simulation, the contact properties between the sample and supports were defined as frictionless, finite sliding and surface-to-surface contact, as shown in Figure 6b.

The material characteristics introduced in the pre-processing stage were partially obtained experimentally, as presented in Table 4. The modeling was performed for linear elastic orthotropic behavior.

To determine the mesh size, a brief analysis was performed to verify the mesh convergence, as presented in Table 5. It can be noted that the progressive refining of the mesh density did not lead to a considerable change in the results (less than 5%); as a result, the mesh size was set to 1 mm in subsequent finite element analyses.

## 3. Results and Discussion

### 3.1. HPLA Behavior to Tensile Stress

**Figure 7:**
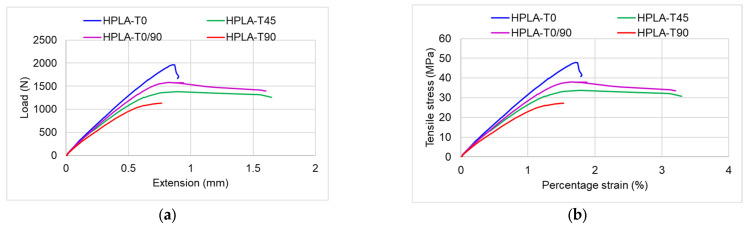
Comparison of HPLA samples with different raster angle orientations subjected to tensile tests: (**a**) load–force curves; (**b**) stress–strain curves.

**Figure 8:**
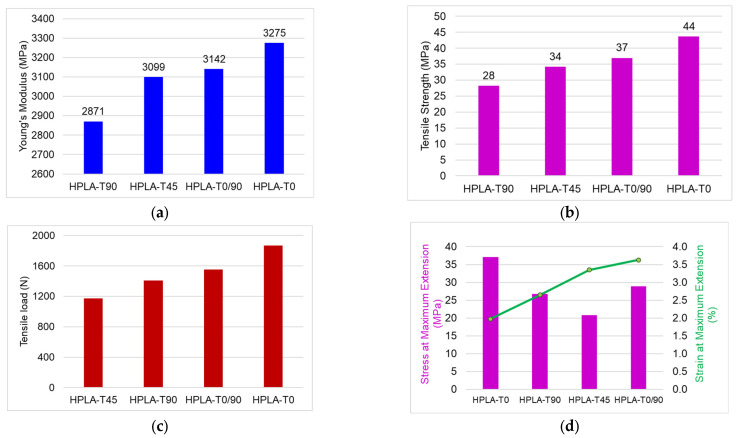
Comparison of different mechanical properties of HPLA samples obtained from tensile tests: (**a**) elasticity modulus; (**b**) tensile strength; (**c**) tensile load; (**d**) stress versus strain.

The characteristic curves obtained for tensile stress for each type of tested sample are presented in Supplementary Materials—Figure S2. The behavior of the samples differs depending on the raster angle orientation; the samples printed with a raster angle of 45° and those with an alternative raster angle of 0/90° show similar behavior in the elastic zone, the yield zone and the plastic zone. The samples printed concentrically and those with a raster angle of 90° have a similar behavior from an elastic–viscoplastic point of view but with different stress intensities. The comparative behavior of the four types of samples, both in terms of the force–displacement curve and in the case of the stress–strain curve, is shown in Figure 7. The variation in mechanical properties (modulus of elasticity, tensile strength, load, and strain) depending on the raster angle orientation is highlighted in Figure 8. It can be observed that samples with concentric raster orientation present the highest values of modulus of elasticity, stress, and maximum force but the lowest value of strain. In terms of modulus of elasticity, samples HPLA-T0/90 and HPLA-T45 present values 4–5% lower than HPLA-T0 and values 12.3% lower than HPLA-T90 (Figure 8a). A greater impact of the raster angle pattern is observed in the case of tensile strength, which reaches maximum values for HPLA-T0 samples (43 MPa average value), with the other samples registering values 15% (HPLA-T0/90), 21% (HPLA-T45) and 35% (HPLA-T90) lower. Compared to the filament strength value reported in the technical sheet of 52 MPa, the printed samples exhibit a reduction in strength by approximately 16–46% (Figure 8b). The maximum breaking load decreases between 1869 and 1174 N, depending on the raster angle orientation (Figure 8c). The variation in strain with raster angle is presented in Figure 8d. The stiffest samples are HPLA-T0 with an average strain value of 1.97%, whereas the HPLA-T0/90 samples exhibit the highest strain at 3.62%.

### 3.2. HPLA Behavior to Bending

**Figure 9:**
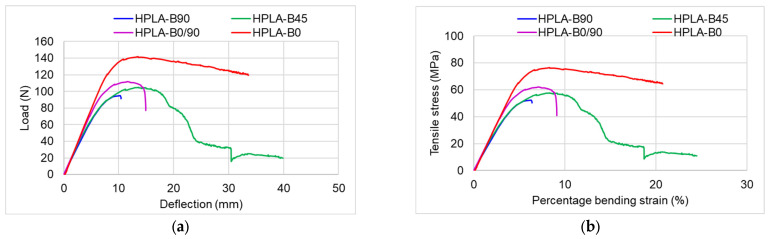
Characteristic curves of HPLA samples with different raster angle orientations, subjected to bending: (**a**) comparison of load–force curves of HPLA samples with different raster orientations; (**b**) comparison of stress–strain curves of HPLA samples with different raster orientations.

The flexural behavior of the samples is highlighted in Figure 9 and in Supplementary Materials—Figure S3 by means of the characteristic curves grouped into the four types of samples tested. The evolution of the samples during bending is influenced by the 3D printing mode, with each category of samples having a specific imprint on the stress–strain curves. The major differences appear in the yield zone and then the plastic zone, followed by sample failure, as can be seen in Figure 9.

**Figure 10:**
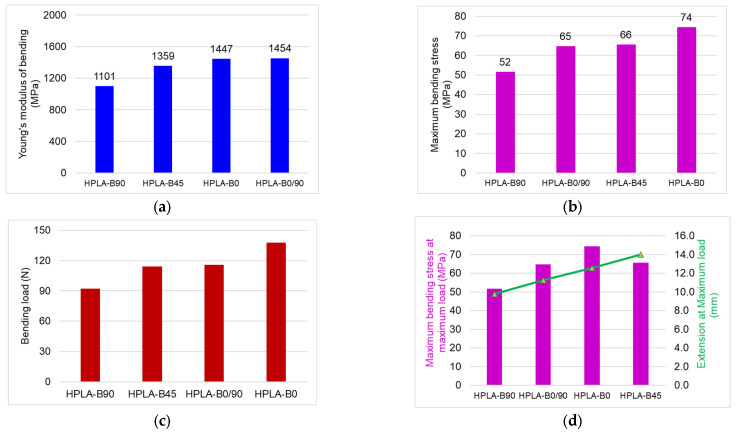
Comparison of different mechanical properties of HPLA samples subjected to bending: (**a**) Young’s Modulus; (**b**) tensile strength; (**c**) tensile load; (**d**) stress versus strain.

Figure 10 presents the main mechanical properties, such as flexural modulus, strength, maximum load, and specific strain. The elastic modulus of the samples varies between 1100 and 1454 MPa, with the most elastic sample being HPLA-B90 and the stiffest one being HPLA-B0/90. Compared to the manufacturer’s specifications, where the flexural modulus is 2490 MPa, the tested samples have flexural modulus values approximately 41–63% lower (Figure 10a). Comparisons of the maximum bending stress of the tested samples are highlighted in Figure 10b, with the highest value being recorded for the HPLA-B0 samples (74 MPa) and the lowest, by approximately 30%, for the HPLA-B90 samples.

The maximum load varies, increasing from 92 N in the case of HPLA-B90 samples, 115 N for HPLA-B0/90 samples, and 114 N for HPLA-B45 samples, with the highest value of 137 N obtained for HPLA-B0 samples (Figure 10c). In Figure 10d, a linear increase in deflection as a function of raster angle orientation can be observed, with minimum values for HPLA-B90 and maximum values for HPLA-B45.

### 3.3. Finite Element Analysis

#### 3.3.1. Simulation of Tensile Behavior

**Figure 11:**
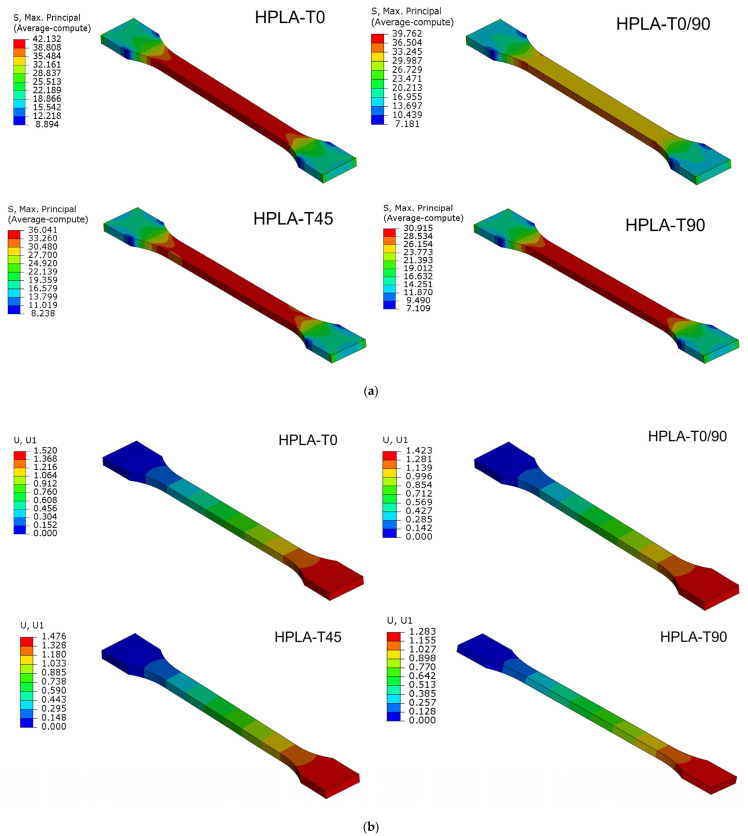
Simulation of tensile behavior: (**a**) maximum tensile stress state; (**b**) displacement state.

**Figure 12:**
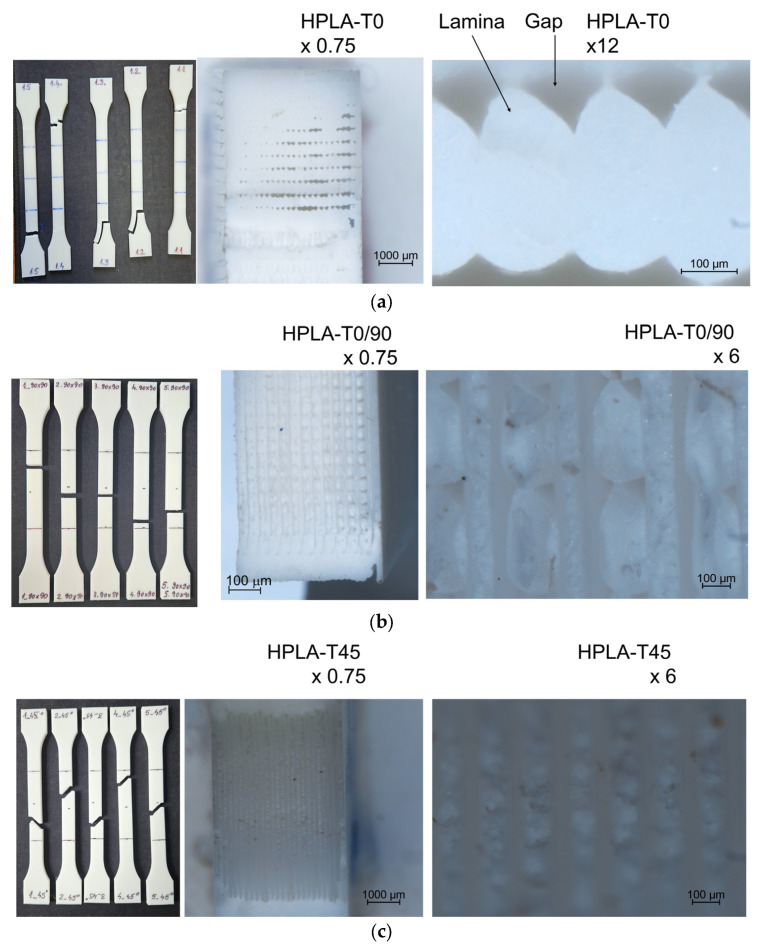

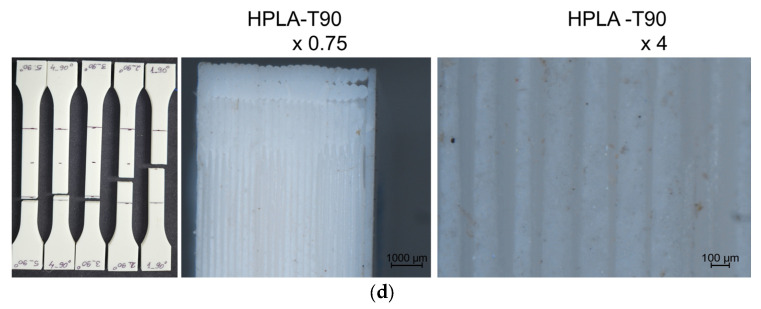
Microscopy images of fractured surfaces after tensile stress: (**a**) HPLA–T0; (**b**) HPLA–T0/90; (**c**) HPLA–T45; (**d**) HPLA–T90.

The comparison between finite element analysis (FEA) and experimental results (EXP) is shown in Table 6. The states of tensile stress and displacements obtained by finite element analysis are presented in Figure 11. It can be observed that between the values obtained experimentally and those determined by finite element analysis, the differences vary between 3 and 10% for normal stresses and between 3 and 20% for elongations. These differences are due to the simulation of the bulk material compared to the real section of the fused deposition modeling orientation of HPLA, which contains air gaps or voids, even if the infill was theoretically 100%, as shown in Figure 12. This phenomenon was studied by [43,44,45], who proposed a set of equations for determining the constitutive properties of an FDM material with cavities, the most affected being the longitudinal elasticity modulus E22 and the shear modulus G12. Using finite elements, ref. [45] analyzed the behavior of unidirectional FFF-printed PLA specimens under tensile loading with different raster angle orientations, highlighting the fact that modeling must consider air gaps and the shape of the printed filament section for the simulation results to have an error less than 5%.

The microscopic images of the fracture areas in the sections of the four types of samples are presented in Figure 12. Taking into account all microstructure features, refs. [46,47,48] proposes a coupled multiscale model to account for the presence of inhomogeneity in 3D-printed parts due to voids of different sizes and shapes resulting from the extrusion processing of polymer parts, as well as the effects of processing parameters on bond adhesion and microstructure, such as filling density and build direction, in the case of ABS filaments.

#### 3.3.2. Simulation of Bending Behavior

**Figure 13:**
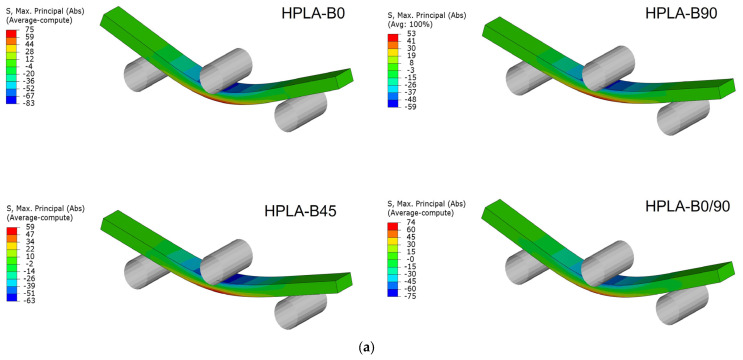

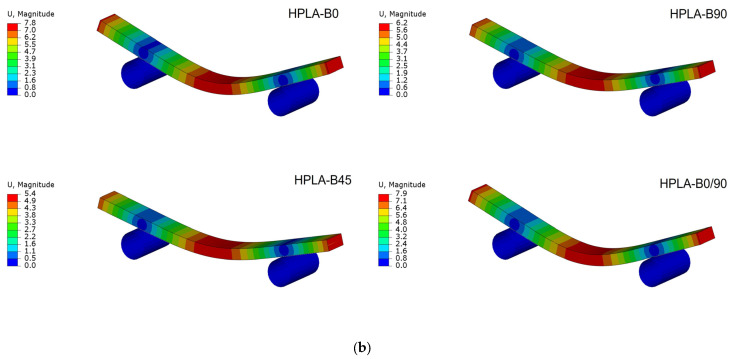
Simulation of bending behavior: (**a**) maximum bending stress state; (**b**) flexural state.

In Figure 13, the states of stress and strain in the case of bending stress are presented. Refs. [47,48] highlighted that explicit microstructural simulation by finite elements (FEs) is necessary to analyze the stress at the interface between the fibers and adjacent layers. Unlike them, the finite element simulation in the present study aimed to analyze the differences between the experimental and numerical results, considering that the samples have the same apparent cross-section as in the real test. In the case of samples subjected to bending, the closest values of the normal stresses are obtained for the concentric pattern; in the case of other orientations, the differences vary between 10 and 14%. The maximum displacements in bending show higher values in the real test by up to 47% (samples with raster angle orientation at 45°) (Table 7).

**Figure 14:**
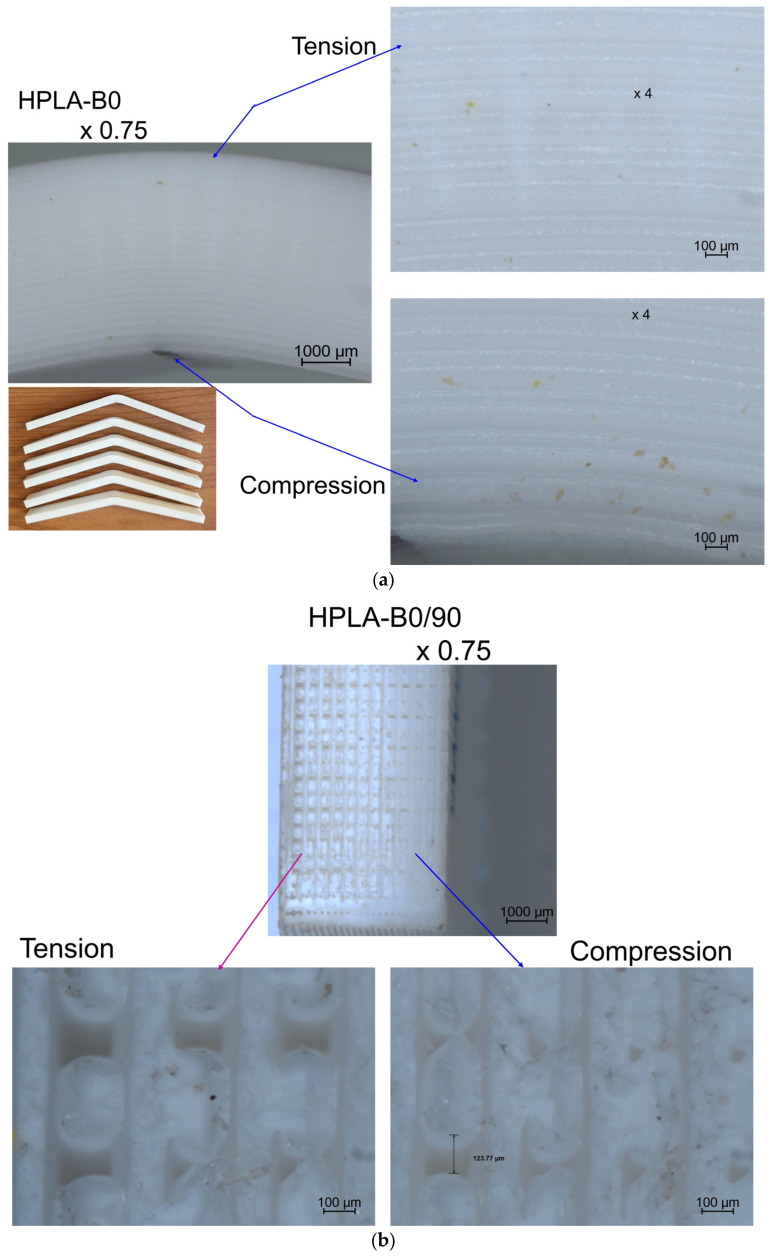

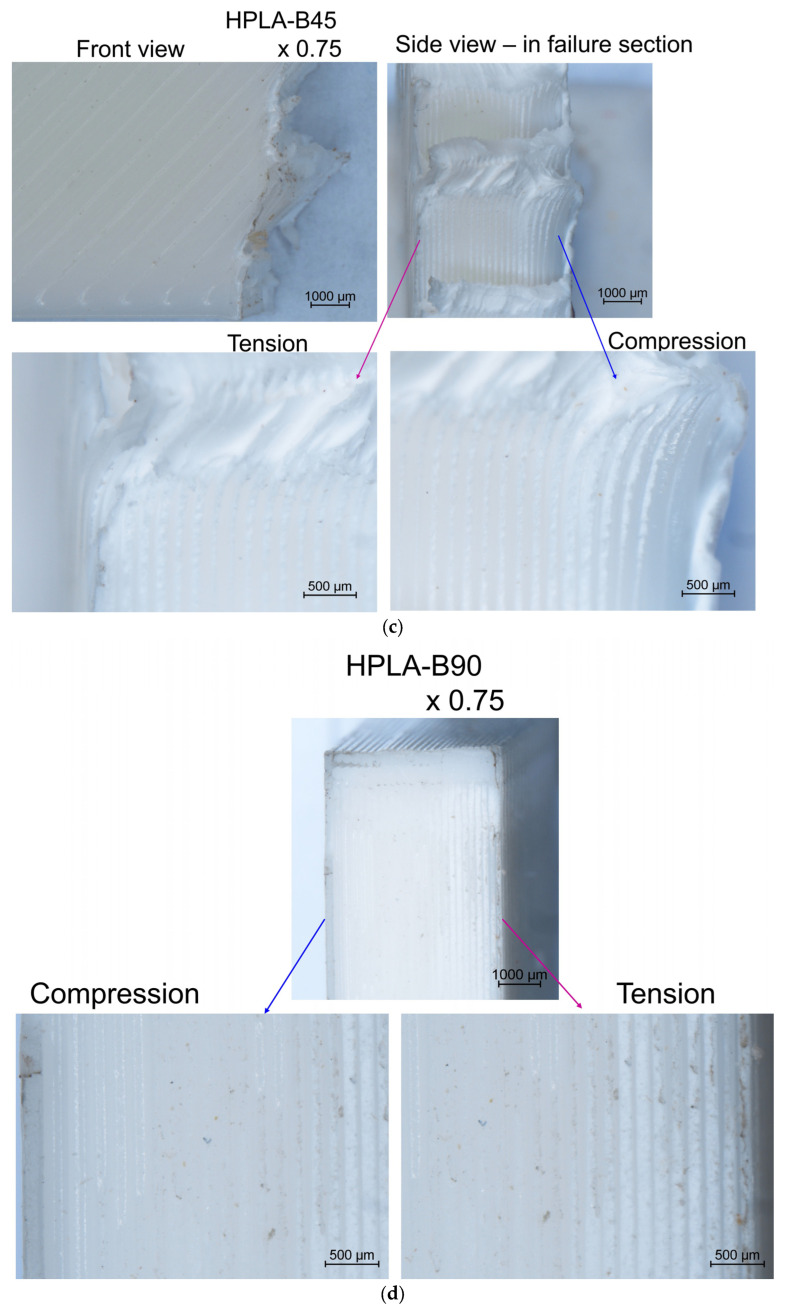
Microscopy images of fracture surfaces after bending stress: (**a**) HPLA-B0; (**b**) HPLA-B0/90; (**c**) HPLA-B45; (**d**) HPLA-B90.

The effect of the bending strain of the samples can be observed in Figure 14. The sample with a concentric pattern deformed in the viscoplastic range until it slipped from the jaws of the testing machine, without breaking. In the other types of samples, in the broken section, the tension and compression zones produced by the bending stress could be observed, as well as the delamination between the layers produced by the shear stress.

### 3.4. Results of Statistical Analysis

The experimental results were statistically analyzed in order to highlight the links between the mechanical characteristics and the 3D printing method. Following verification with the Shapiro–Wilk test, it was found that the batch of samples subjected to investigation is quite homogeneous in terms of most mechanical properties, except for flexural, bending deformations, and mechanical work (B_Strain, B_Work and B_extension), where the coefficients of variation are high, as shown in the Supplementary Materials—Table S4. The degree of dispersion around the mean of the mechanical parameters is higher in bending than in tension (the average coefficients of variation are 28.6% and 18.2%, respectively). All mechanical parameters differ in relation to raster angle. From the matrix of Spearman’s simple correlation coefficients, it was found that the intensity of the links between the indices of the same mechanical stress (tension and bending, respectively) is as strong as the intensity of the links between the two stresses (Table 8).

**Figure 15:**
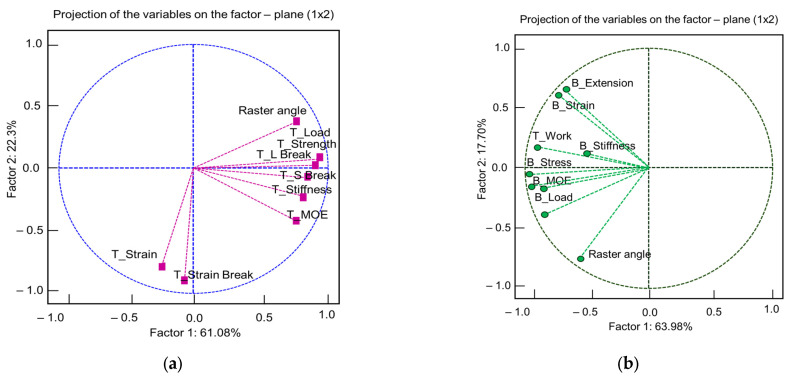
PCA of the analyzed variables: (**a**) tensile; (**b**) bending.

Principal component analysis (PCA) highlighted the most influential mechanical variables in relation to raster angle (Figure 15). It can be seen that the results are very different in the case of bending compared to tension. For tensile loading, six principal components were extracted, with the first two explaining together 84% of the total variance. In tension, the polarization of the variables into two groups is noticeable. The first group is also closely related to the raster angle. The most influential variables in the group are T-Strength, T_load, and T-L Break. The second group only has two variables, T_Strain Break and T-Strain. The two groups are divergent. The first principal component, which explains 61% of the total variance, is oriented in the direction of T_strength (factor loadings = +0.93) (Figure 15a). The second principal component is oriented in the direction of T_Strain Break. In Figure 15b, for the bending test, the variables are distributed more on the negative axis of the first factor, which explains 64% of the variation. The axis is oriented in the direction of B_Stress and B_Load (loading factor −0.97). The secondary axis for bending is defined by the raster angle (loading factor −0.74). At the positive side of the axis is B_strain (loading factor +0.60). The bending indices do not respond as quickly to changes in raster angle as the tensile indices (Figure 15b).

Discriminant analysis was performed with six independent variables, as shown by PCA and the grouping variable (the raster angle). The results of discriminant analysis are presented in Table 9. The discriminant model is statistically significant: F (18, 31) = 16.775, *p* ˂ 0.0001, meaning that the variables in the model significantly differentiate the raster angle. Thus, the most important variable in the model, which explains the differences between the raster angles, is the tensile strain at break (*p* ˂ 0.0001), followed by the bending stress.

**Figure 16:**
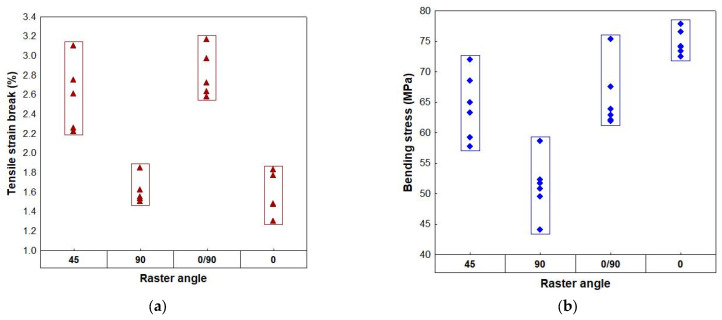
Variability plot as a function of raster angle: (**a**) tensile strain at break; (**b**) bending stress.

From an application point of view, the obtained results highlight the fact that the raster angle orientation affects the deformation and crack propagation mechanism more than the maximum stress. For the variables with the highest ability to discriminate—tensile strain and bending stress—their values were stratified in relation to these classes, as can be seen in Figure 16.

## 4. Conclusions

In this study, the behavior of 3D-printed HPLA samples with different raster angles of orientation was analyzed, and the main elastic characteristics were determined under tensile and three-point bending stress. These results were statistically analyzed to establish the variance of the parameters that influence these quantities and the correlations between them. Subsequently, numerical simulation of the samples was performed using the experimentally determined characteristics, with the results highlighting the difference between the two investigation methods. The most important conclusions are as follows:

- Raster angle is the parameter that most significantly influences the behavior of the samples in both types of loading.
- The differences between raster angles are mainly explained by the tensile strain at break, followed by the bending stress.
- The closest connection between raster angle orientation and the type of load is observed under tension.
- The difference between the simulation results and the tests under tension load is within a maximum of 10%. Compared to the bending results, where the differences are greater, the finite element analysis method requires a different approach to modeling the structures.
- The voids inside the printed material, even under the conditions of a 100% infill printing strategy, are responsible for the differences between experiment and simulation.
- The plastic deformations that occur inside the structure are visible with the optical microscope and correlate with the experimental results.
- In future studies, the authors propose a simulation of the samples at the microscale in such a way as to consider the distribution of the material in the section.

## Figures and Tables

**Table 1:** FDM printer setup parameters.

Parameter	Units	Value
Hyper PLA filament diameter	(mm)	1.75
Nozzle diameter	(mm)	0.4
Layer thickness	(mm)	0.2 (1st layer); 0.16 (24 layers)
Number of outer walls	(-)	2
Nozzle temperature	(°C)	220
Bed temperature	(°C)	60
Layer resolution		0.012
Infill density	(%)	100
Infill pattern	(-)	Aligned rectilinear
Raster orientation angle	(°)	0; 45; 90; 0/90
Printed speed	(mm/s)	300

**Table 2:** Geometrical characteristics of tensile samples correlated with raster angle.

Raster Angle	Sample Code	Thickness (mm)	Gauge Length (mm)	No. of Samples
Concentric	HPLA-T0	4	50	5
45°	HPLA-T45	4	50	5
90°	HPLA-T90	4	50	5
0°/90°	HPLA-T0/90	4	50	5

**Table 3:** Geometrical characteristics of bending samples correlated with raster angle.

Raster Angle	Sample Code	Type of Sample Test	Thickness (mm)	Gauge Length (mm)	No. of Samples
Concentric	HPLA-B0	Bending (ISO 178:2019)	4	64	6
45°	HPLA-B45	Bending (ISO 178:2019)	4	64	6
90°	HPLA-B90	Bending (ISO 178:2019)	4	64	6
0°/90°	HPLA-B0/90	Bending (ISO 178:2019)	4	64	6

**Table 4:** Geometrical characteristics of samples correlated to raster angle orientation and local coordinate systems.

Young’s Modulus (MPa)			Shear Modulus (MPa)			Poisson Coefficient			Density (kg/m^3^)
E1	E2	E3	G12	G23	G31	$\vartheta_{12}$	$\vartheta_{23}$	$\vartheta_{31}$	ρ
3275	2870	2870	1000	1146	1000	0.428	0.435	0.258	1240

**Table 5:** Mesh convergence analysis.

	Mesh Size			
	0.5 mm	1.00 mm	1.5 mm	4 mm
	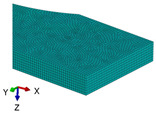	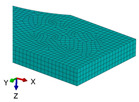	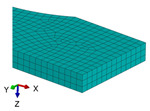	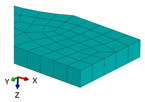
Stress (MPa)	31.50	30.92	30.62	29.49
Displacement (mm)	1.283	1.283	1.283	1.282

**Table 6:** Comparison of experimental and simulated results of tensile behavior.

Type of Sample	Tensile Stress (MPa)		Differences (%)	Elongation (mm)		Differences (%)
	FEA	EXP		FEA	EXP	
HPLA-T0	43.64	42.13	+3.46	1.52	1.21	−20.39
HPLA-T0/90	39.76	36.96	+7.58	1.42	1.60	+12.67
HPLA-T45	36.04	34.20	+5.38	1.48	1.59	+7.43
HPLA-T90	30.91	28.24	+9.47	1.28	1.32	−3.13

**Table 7:** Comparison of experimental and simulated results of bending behavior.

Type of Sample	Bending Stress (MPa)		Differences (%)	Flexural (mm)		Differences (%)
	FEA	EXP		FEA	EXP	
HPLA-B0	75	74	+1.33	7.9	7.8	+1.26
HPLA-B0/90	74	64	+13.13	7.8	6.9	+11.53
HPLA-B45	59	65	−10.17	5.7	8.4	−47.36
HPLA-B90	53	51	+3.77	5.7	9.7	−23.54

**Table 8:** Matrix of Spearman simple correlation coefficients between experimentally determined mechanical parameters.

Mechanical Parameters	Simple Correlation Coefficients (* Statistically Significant Values (*p* ≤ 0.5))																		
	T_Stiffness	T_MOE	T_Load	T_Stress	T_Extension	T_Strain	T_L Break	T_S Break	T_Strain Break	T_Strength	B_Stiffness	B_MOE	B_Load	B_Stress	B_Extension	B_Rigidity	B_Strain	B_Work	B_Extension
T_Stiffness		0.885 *	0.805 *	0.783 *	−0.037	−0.037	0.671 *	0.586 *	0.102	0.783 *	0.210	0.777 *	0.803 *	0.790 *	0.399	0.859 *	0.487 *	0.692 *	0.477 *
T_MOE			0.642 *	0.725 *	0.132	0.132	0.648 *	0.682 *	0.264	0.725 *	0.161	0.689 *	0.697 *	0.702 *	0.429	0.735 *	0.463 *	0.580 *	0.458 *
T_Load				0.975*	−0.282	−0.282	0.887 *	0.784 *	−0.091	0.975 *	0.345	0.823 *	0.946 *	0.920 *	0.492 *	0.904 *	0.612 *	0.855 *	0.599 *
T_Stress					−0.219	−0.219	0.926 *	0.876 *	−0.020	1.000 *	0.339	0.822 *	0.944 *	0.925 *	0.529 *	0.890 *	0.628 *	0.844 *	0.616 *
T_Extension						1.000*	−0.251	−0.163	0.741 *	−0.219	−0.231	−0.028	−0.216	−0.158	0.071	−0.038	−0.188	−0.436	−0.173
T_Strain							−0.251	−0.163	0.741 *	−0.219	−0.231	−0.028	−0.216	−0.158	0.071	−0.038	−0.188	−0.436	−0.173
T_L Break								0.969 *	−0.063	0.926 *	0.308	0.780 *	0.808 *	0.800 *	0.473 *	0.812 *	0.591 *	0.772 *	0.581 *
T_S Break									0.017	0.876 *	0.277	0.714 *	0.734 *	0.736 *	0.481 *	0.726 *	0.568 *	0.698 *	0.561 *
T_Strain Break										−0.020	−0.206	0.095	−0.043	0.018	0.293	0.044	0.052	−0.275	0.064
T_Strength											0.339	0.822*	0.944 *	0.925 *	0.529 *	0.890 *	0.628 *	0.844 *	0.616 *
B_Stiffness												0.356	0.342	0.366 *	0.249	0.151	0.463 *	0.521 *	0.456 *
B_MOE													0.852 *	0.884 *	0.543 *	0.849 *	0.543 *	0.731 *	0.541 *
B_Load														0.991*	0.606 *	0.881 *	0.646 *	0.876 *	0.636 *
B_Stress															0.671 *	0.855 *	0.691 *	0.876 *	0.685 *
B_Extension																0.428	0.886 *	0.684 *	0.893 *
B_Rigidity																	0.431	0.699 *	0.421
B_Strain																		0.843 *	1.000 *
B_Work																			0.833 *
B_Extension																			

**Table 9:** Results of discriminant analysis.

Variables	Wilks’ Lambda	Partial Lambda	F-Remove	*p*-Level	Tolerance	1 − Tolerance
T_Load	0.002208	0.576913	2.68900	0.097752	0.422314	0.577686
T_L Break	0.001500	0.849278	0.65072	0.598822	0.218575	0.781425
T_Strain Break *	0.009007	0.141427	22.25954	0.000056	0.636765	0.363235
T_Strength	0.001596	0.798317	0.92633	0.460268	0.165138	0.834862
B_Load	0.002416	0.527353	3.28630	0.062085	0.057407	0.942593
B_Stress *	0.002505	0.508599	3.54269	0.051609	0.060345	0.939655

* The most important variables in the model.

## Data Availability

The original contributions presented in this study are included in the article. Further inquiries can be directed to the corresponding author.

## Supplementary Materials

The following supporting information can be downloaded at https://www.mdpi.com/article/10.3390/polym18050624/s1, Figure S1. The sample overview and meshing: (a) design of tensile samples; (b) bending sample; Figure S2. Characteristic curves of HPLA samples with different raster orientations subjected to tensile test: (a) stress–strain curves for HPLA-T0; (b) stress–strain curves for HPLA-T45; (c) stress–strain curves for HPLA-T90; (d) stress–strain curves for HPLA-T0/90; Figure S3. Characteristic curves of HPLA samples with different raster orientations subjected to bending: (a) stress–strain curves for HPLA-B0; (b) stress–strain curves for HPLA-B90; (c) stress–strain curves for HPLA-B45; (d) stress–strain curves for HPLA-B0/90; Table S1. The most used types of filaments and their mechanical properties in 3D printing technology by FFF or/and FDM; Table S2. Sequences from the printing program with highlighting of the printing parameter settings; Table S3. HPLA Filament Technical Data Sheet; Table S4. Descriptive statistics of the examined properties.

## Abbreviations

The following abbreviations are used in this manuscript:

CAD	Computer-Aided Design
CAM	Manufacturing-Aided Design
CAE	Engineering-Aided Design
AM	Additive Manufacturing
FFF	Fused Filament Fabrication
FDM	Fused Deposition Modeling
PLA	Polylactic Acid
ABS	Acrylonitrile Butadiene Styrene
CFF	Carbon Fiber Filament
PA	Aka Polyamide
FLEX	Flexible Filaments
HIPS	High-Impact Polystyrene
PVA	Polyvinyl Alcohol Filament
PETG	Polyethylene Terephthalate Glycol-Modified
TPE	Thermoplastic Elastomers Filament
PC	Polycarbonate Filament
HPLA	Hyper Polylactic Acid
PCA	Principal Component Analysis
T	Tensile Test
B	Bending Test
T_Stiffness	Tensile Stiffness
T_MOE	Young’s Modulus to tensile
T_Load	Load at Maximum Load
T_Stress	Stress at Maximum Load
T_Extension	Extension at Maximum Load
T_Strain	Percentage Strain at Maximum Extension
T_L Break	Load at Break
T_S Break	Stress at Break
T_Strain Brake	Percentage Strain at Break
T_Strength	Tensile Strength
B_Stiffness	Bending stiffness
B_MOE	Young’s Modulus to bending
B_Load	Load at Maximum Load
B_Stress	Stress at Maximum Load
B_Extension	Extension at Maximum Load
B_Rigidity	Flexural Rigidity
B_Strain	Maximum Bending Strain at Maximum Extension
B_Work	Work to Maximum Extension

## References

[1] Horvath J., Cameron R. (2020). Why Use a 3D Printer?. Mastering 3D Printing.

[2] Tao F., Ma X., Liu W., Zhang C. (2024). Digital Engineering: State-of-the-art and perspectives. Digit. Eng..

[3] Islam A., Mobarak H., Rimon I.H., Al Mahmud Z., Ghosh J., Ahmed M.S., Hossain N. (2024). Additive manufacturing in polymer research: Advances, synthesis, and applications. Polym. Test..

[4] Bharat N., Jain R., Bose P.S.C., Sharma V.S., Dixit U.S., Gupta A., Verma R., Sharma V. (2024). A Comprehensive Overview on Additive Manufacturing Processes: Materials, Applications, and Challenges. Machining and Additive Manufacturing.

[5] Pozorski Z., Andrzejewski J. (2025). Experimental determination of mechanical properties of 3D printed PLA. Methodology for testing orthotropic materials. Polym. Test..

[6] Cojocaru V., Frunzaverde D., Miclosina C.-O., Marginean G. (2022). The Influence of the Process Parameters on the Mechanical Properties of PLA Specimens Produced by Fused Filament Fabrication—A Review. Polymers.

[7] Tiismus H., Kallaste A., Vaimann T., Rassõlkin A. (2022). State of the art of additively manufactured electromagnetic materials for topology optimized electrical machines. Addit. Manuf..

[8] Altıparmak S.C., Yardley V.A., Shi Z., Lin J. (2022). Extrusion-based additive manufacturing technologies: State of the art and future perspectives. J. Manuf. Process..

[9] Spoerk M., Gonzalez-Gutierrez J., Sapkota J., Schuschnigg S., Holzer C. (2018). Effect of the printing bed temperature on the adhesion of parts produced by fused filament fabrication. Plast. Rubber Compos. Macromol. Eng..

[10] Ahn S.-H., Montero M., Odell D., Roundy S., Wright P.K. (2002). Anisotropic material properties of fused deposition modeling ABS. Rapid Prototyp. J..

[11] Ferreira R.T.L., Amatte I.D., Dutra T.A., Burger D. (2017). Experimental characterization and micrography of 3D printed PLA and PLA reinforced with short carbon fibers. Compos. Part B Eng..

[12] Sandu I.-L., Stan F., Fetecau C. (2023). Mechanical Recycling of Ethylene-Vinyl Acetate/Carbon Nanotube Nanocomposites: Processing, Thermal, Rheological, Mechanical and Electrical Behavior. Polymers.

[13] Kadhum A.H., Al-Zubaidi S., Abdulkareem S.S. (2023). Effect of the Infill Patterns on the Mechanical and Surface Characteristics of 3D Printing of PLA, PLA+ and PETG Materials. ChemEngineering.

[14] Park Y.-E., Lee S. (2024). Characterization of PLA/LW-PLA Composite Materials Manufactured by Dual-Nozzle FDM 3D-Printing Processes. Polymers.

[15] Dev S., Srivastava R. (2021). Effect of infill parameters on material sustainability and mechanical properties in fused deposition modelling process: A case study. Prog. Addit. Manuf..

[16] Cañero-Nieto J.M., Campo-Campo R.J., Díaz-Bolaño I., Ariza-Echeverri E.A., Deluque-Toro C.E., Solano-Martos J.F. (2025). Infill pattern strategy impact on the cross-sectional area at gauge length of material extrusion 3D printed polylactic acid parts. J. Intell. Manuf..

[17] Patel K.S., Shah D.B., Joshi S.J., Aldawood F.K., Kchaou M. (2024). Effect of process parameters on the mechanical performance of FDM printed carbon fiber reinforced PETG. J. Mater. Res. Technol..

[18] Liu X., Zhang M., Li S., Si L., Peng J., Hu Y. (2017). Mechanical property parametric appraisal of fused deposition modeling parts based on the gray Taguchi method. Int. J. Adv. Manuf. Technol..

[19] Popescu D., Zapciu A., Amza C., Baciu F., Marinescu R. (2018). FDM process parameters influence over the mechanical properties of polymer specimens: A review. Polym. Test..

[20] Orellana-Barrasa J., Ferrández-Montero A., Boccaccini A.R., Ferrari B., Pastor J.Y. (2022). The Mechanical, Thermal, and Chemical Properties of PLA-Mg Filaments Produced via a Colloidal Route for Fused-Filament Fabrication. Polymers.

[21] Caminero M., Chacón J., García-Plaza E., Núñez P., Reverte J., Bécar J. (2019). Additive Manufacturing of PLA-Based Composites Using Fused Filament Fabrication: Effect of Graphene Nanoplatelet Reinforcement on Mechanical Properties, Dimensional Accuracy and Texture. Polymers.

[22] Kantaros A., Katsantoni M., Ganetsos T., Petrescu N. (2025). The Evolution of Thermoplastic Raw Materials in High-Speed FFF/FDM 3D Printing Era: Challenges and Opportunities. Materials.

[23] El-Deeb I.S., Petrov M.A., Grabowik C., Esmael E.G., Rashad M., Ebied S., Burduk A., Batako A.D.L., Machado J., Wyczółkowski R., Dostatni E., Rojek I. (2024). Mechanical Properties of PLA Printed Samples in Different Printing Directions and Orientations Using Fused Filament Fabrication, Part 1: Methodology. Intelligent Systems in Production Engineering and Maintenance III. ISPEM 2023.

[24] El-Deeb I.S., Petrov M.A., Grabowik C., Esmael E.G., Rashad M., Ebied S., Burduk A., Batako A.D.L., Machado J., Wyczółkowski R., Dostatni E., Rojek I. (2024). Mechanical Properties of PLA Printed Samples in Different Printing Directions and Orientations Using Fused Filament Fabrication, Part 2: Experimental Research. Intelligent Systems in Production Engineering and Maintenance III. ISPEM 2023.

[25] Calì L.M., Pascoletti G., Gaeta M., Milazzo G., Ambu R. (2020). New filaments with natural fillers for FDM 3D printing and their applications in biomedical field. Procedia Manuf..

[26] Lohar D.V., Nikalje A.M., Damle P.G. (2022). Development and testing of hybrid green polymer composite (HGPC) filaments of PLA reinforced with waste bio fillers. Mater. Today Proc..

[27] Arockiam A.J., Subramanian K., Padmanabhan R.G., Selvaraj R., Bagal D.K., Rajesh S. (2022). A review on PLA with different fillers used as a filament in 3D printing. Mater. Today Proc..

[28] Vălean C., Stoia D.I., Opriș C., Linul E. (2024). Effect of Fillers on Mechanical Properties of FDM printed PLA Components. Procedia Struct. Integr..

[29] Plamadiala I., Croitoru C., Pop M.A., Roata I.C. (2025). Enhancing Polylactic Acid (PLA) Performance: A Review of Additives in Fused Deposition Modelling (FDM) Filaments. Polymers.

[30] Naveed N. (2021). Investigating the Material Properties and Microstructural Changes of Fused Filament Fabricated PLA and Tough-PLA Parts. Polymers.

[31] Gao G., Xu F., Xu J., Tang G., Liu Z. (2022). A Survey of the Influence of Process Parameters on Mechanical Properties of Fused Deposition Modeling Parts. Micromachines.

[32] (2025). Plastics—Determination of Tensile Properties. Part 2: Test Conditions for Moulding and Extrusion Plastics.

[33] (2025). Plastics—Determination of Tensile Properties—Part 1: General Principles.

[34] Es-Said O., Foyos J., Noorani R., Mendelson M., Marloth R., Pregger B. (2000). Effect of layer orientation on mechanical properties of rapid prototyped samples. Mater. Manuf. Process..

[35] Rodríguez-Panes A., Claver J., Camacho A. (2018). The influence of manufacturing parameters on the mechanical behaviour of pla and abs pieces manufactured by fdm: A comparative analysis. Materials.

[36] Somireddy M., De Moraes D.A., Czekanski A. Flexural behavior of fdm parts: Experimental, analytical and numerical study. Proceedings of the 28th Annual International Solid Freeform Fabrication Symposium.

[37] Vaezi M., Chua C.K. (2011). Effects of layer thickness and binder saturation level parameters on 3D printing process. J. Adv. Manuf. Technol..

[38] Qayyum H., Hussain G., Sulaiman M., Hassan M., Ali A., Muhammad R., Wei H., Shehbaz T., Aamir M., Altaf K. (2022). Effect of Raster Angle and Infill Pattern on the In-Plane and Edgewise Flexural Properties of Fused Filament Fabricated Acrylonitrile–Butadiene–Styrene. Appl. Sci..

[39] Stanciu M.D., Drăghicescu H.T., Roșca I.C. (2021). Mechanical Properties of GFRPs Exposed to Tensile, Compression and Tensile–Tensile Cyclic Tests. Polymers.

[40] Stanciu M.D., Nastac S.M., Tesula I. (2022). Prediction of the Damage Effect on Fiberglass-Reinforced Polymer Matrix Composites for Wind Turbine Blades. Polymers.

[41] (2019). Plastics—Determination of Flexural Properties.

[42] (2007). STATISTICA.

[43] Sánchez González C., Pérez Jiménez A., Malvé M., Díaz Jiménez C. (2025). Effect of Annealing on the Mechanical Properties of Composites of PLA Mixed with Mg and with HA. Polymers.

[44] Li L., Sun Q., Bellehumeur C., Gu P. (2002). Composite Modeling and Analysis for Fabrication of FDM Prototypes with Locally Controlled Properties. J. Manuf. Process..

[45] Seibel S., Kiendl J. (2025). A finite element approach for modelling the fracture behaviour of unidirectional FFF-printed parts. Prog. Addit. Manuf..

[46] Sharafi S., Santare M.H., Gerdes J., Advani S.G. (2022). A multiscale modeling approach of the Fused Filament Fabrication process to predict the mechanical response of 3D printed parts. Addit. Manuf..

[47] Ait Benaissa H., Zaghar H., Moujibi N., Sossey-Alaoui I. (2023). Prediction and examination of the impact of the raster angle on the orthotropic elastic response of 3D-printed objects using a novel homogenization strategy based on the real clustering of RVEs. Int. J. Adv. Manuf. Technol..

[48] Gonabadi H., Chen Y., Yadav A., Bull S. (2022). Investigation of the effect of raster angle, build orientation, and infill density on the elastic response of 3D printed parts using finite element microstructural modeling and homogenization techniques. Int. J. Adv. Manuf. Technol..

