# Supplementary material for: Experimental and Numerical Investigation of Mechanical Properties of Hyper Polylactic Acid (HPLA)
Source: Polymers (Basel). 2026 Mar 3;18(5):624. doi: 10.3390/polym18050624 (PMC12987187; doi:10.3390/polym18050624)
Supplement: Supplementary file 1 [file polymers-18-00624-s001.zip › polymers-4143493-supplementary.pdf]

Supplementary Materials

Experimental and Numerical Investigation of Mechanical Properties of Hyper Polylactic Acid (HPLA)

Mariana Domnica Stanciu, Horațiu Drăghicescu Teodorescu, Ionuț Teșulă, Sergiu Valeriu Georgescu and Florin Dinulică

Table S1. The most used types of filaments and their mechanical properties in 3D printing technology by FFF or/and FDM.

Type of filament polymer	Notation	Tensile Strength (MPa)	Flexural Modulus (MPa)	Print	Type of filament polymer
Polylactic Acid	PLA	65	3800	130–180	50
Acrylonitrile Butadiene Styrene	ABS	40	1600–2400	220–240	95–110
Carbon Fiber Filament	CF	46	5260	220–240	100–110
Nylon or Aka Polyamide	PA	40–85	800–2000	225–265	70–90
Flexible Filaments	FLEX	40	9500	210–230	60
High Impact Polystyrene	HIPS	32	1500–3000	230–245	100–115
Polyvinyl Alcohol Filament	PVA	78	-	185–200	45–60
Polyethylene Terephthalate Glycol-modified	PETG	53	2200	230–250	75–90
Thermoplastic Elastomers Filament	TPE	32	3600	-	-
Polycarbonate Filament	PC	72	2200–2500	260–310	80–120

Table S2. Sequences from the printing program with highlighting of the printing parameter settings

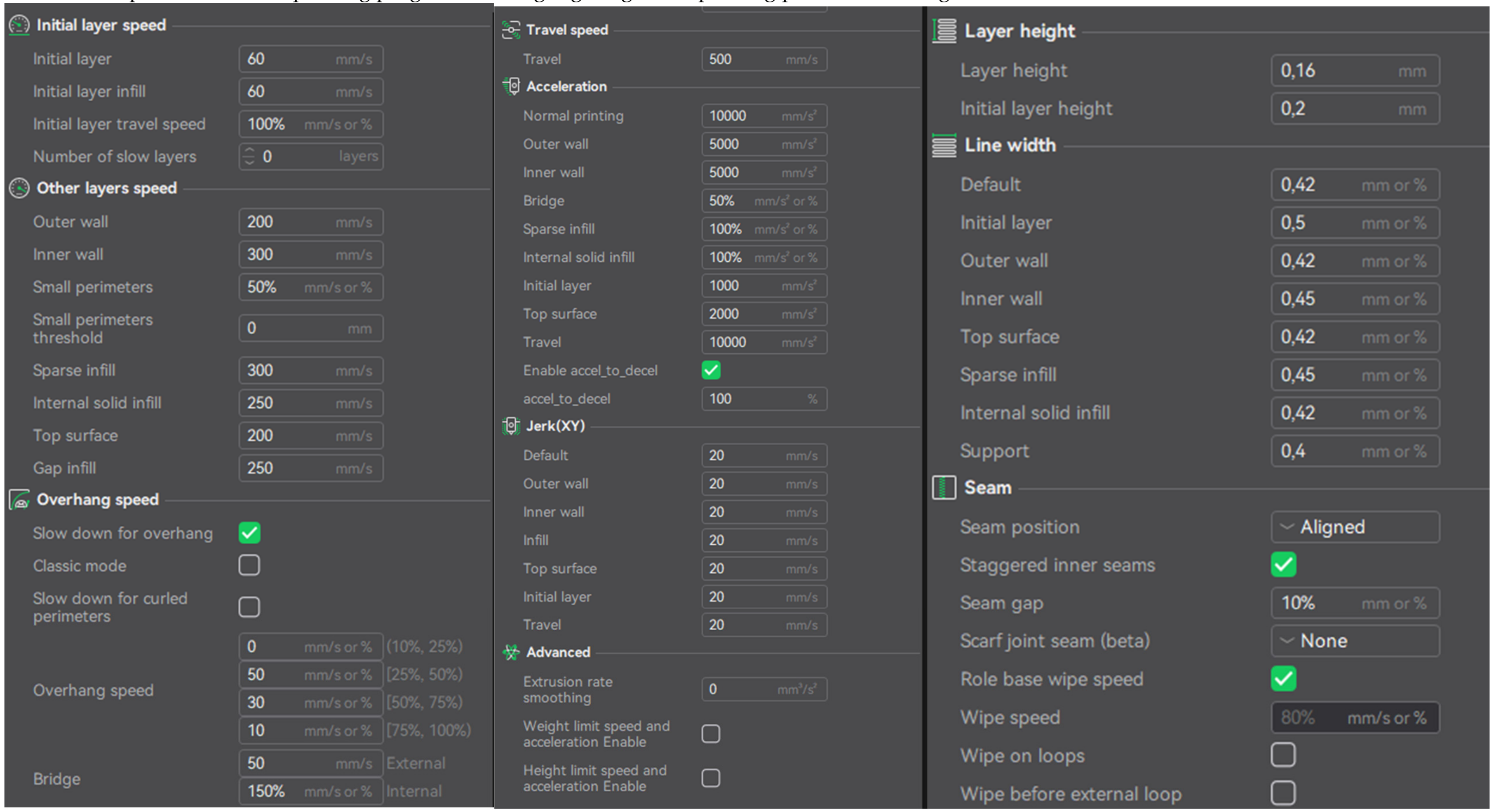

Table S3. HPLA Filament Technical Data Sheet.

Parameter	Units	Value
Density	(g/cm3)	1.24
Glass transition temperature	(°C)	62
Tensile strength (ISO 527)	(MPa)	52
Tensile modulus (ISO 527)	(MPa)	1146
Elongation at break (ISO 527)	(%)	6.3
Bending strength (ISO 178)	(MPa)	92
Bending modulus (ISO 178)	(MPa)	2490
Charpy impact strength	(kJ/m^2)	8.83

Table S4. Descriptive statistics of the examined properties.

Variable	Value	Mean Confidence		Median	Minim	Maxim	Variation coefficient (%)	Normality*	The meaning of the differences between raster angle**
T_Stiffness ($x10^3$)	2595.717	2505.581	2685.853	2255.989	2620.077	2874.192	7.420	YES	**< 0.001**
T_Load	1.501	1.377	1.625	1.133	1.506	1.966	17.670	YES	**< 0.001**
T_Stress	35.763	32.978	38.547	27.235	35.966	47.878	16.638	YES	**< 0.001**
T_Extension	1.449	1.242	1.656	0.780	1.590	2.252	30.525	YES	0.004
T_Strain	2.899	2.485	3.313	1.560	3.179	4.504	30.525	YES	0.004
T_L Break	1.375	1.289	1.460	1.133	1.361	1.799	13.216	NO	0.040
T_S Break	32.807	30.759	34.855	27.235	32.351	43.127	13.337	NO	0.010
T_Strain Break	2.148	1.857	2.439	1.306	2.038	3.171	28.941	NO	0.002
T_Strength	35.763	32.978	38.547	27.235	35.966	47.878	16.638	YES	**< 0.001**
B_Stiffness ($x10^3$)	14.221	12.456	15.483	1.461	14.452	17.022	23.152	NO	0.001
B_MOE	1350.769	1285.589	1377.649	1114.027	1345.383	1504.251	7.386	YES	**< 0.001**
B_Load	0.115	0.105	0.122	0.080	0.113	0.144	16.250	YES	**< 0.001**
B_Stress	63.946	58.250	67.277	44.102	63.035	77.780	15.364	YES	**< 0.001**
B_Extension	12.093	11.268	12.740	9.481	12.068	14.368	13.102	YES	**< 0.001**
B_Rigidity	0.081	0.077	0.084	0.066	0.080	0.093	9.454	YES	**< 0.001**
B_Strain	16.374	12.533	20.316	6.118	19.530	25.172	50.625	NO	**< 0.001**
B_Work	2183.166	1441.423	2875.503	518.925	1726.048	4706.340	70.981	NO	**< 0.001**
B_Extension	26.850	20.510	33.272	10.060	31.811	40.624	50.699	NO	0.002

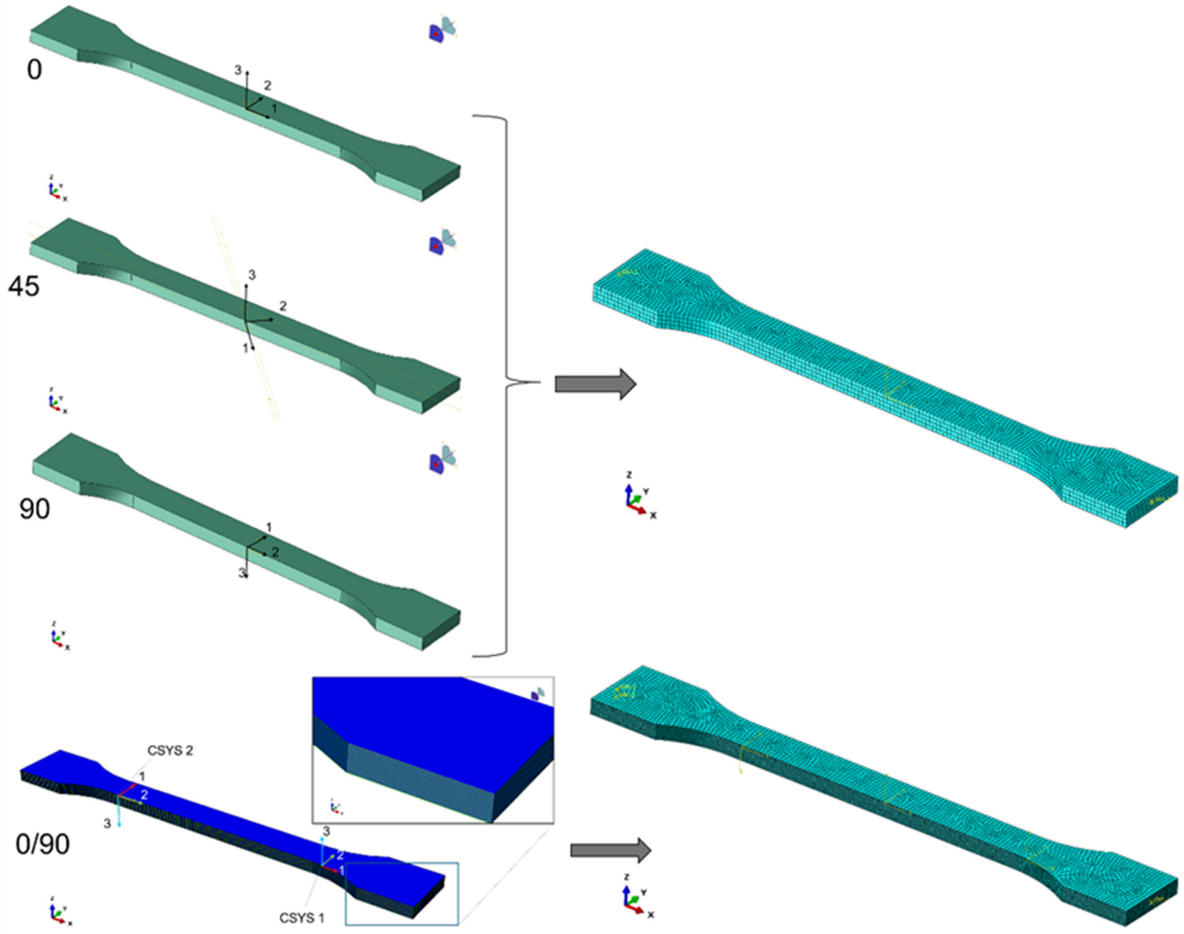

(a)

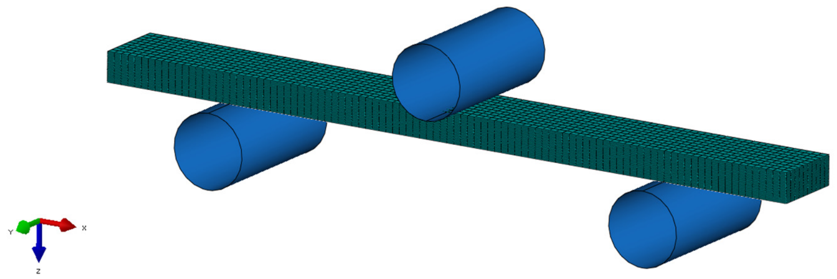

(b)

Figure S1. The samples overview and meshing: (**a**) design of tensile samples; (**b**) bending sample.

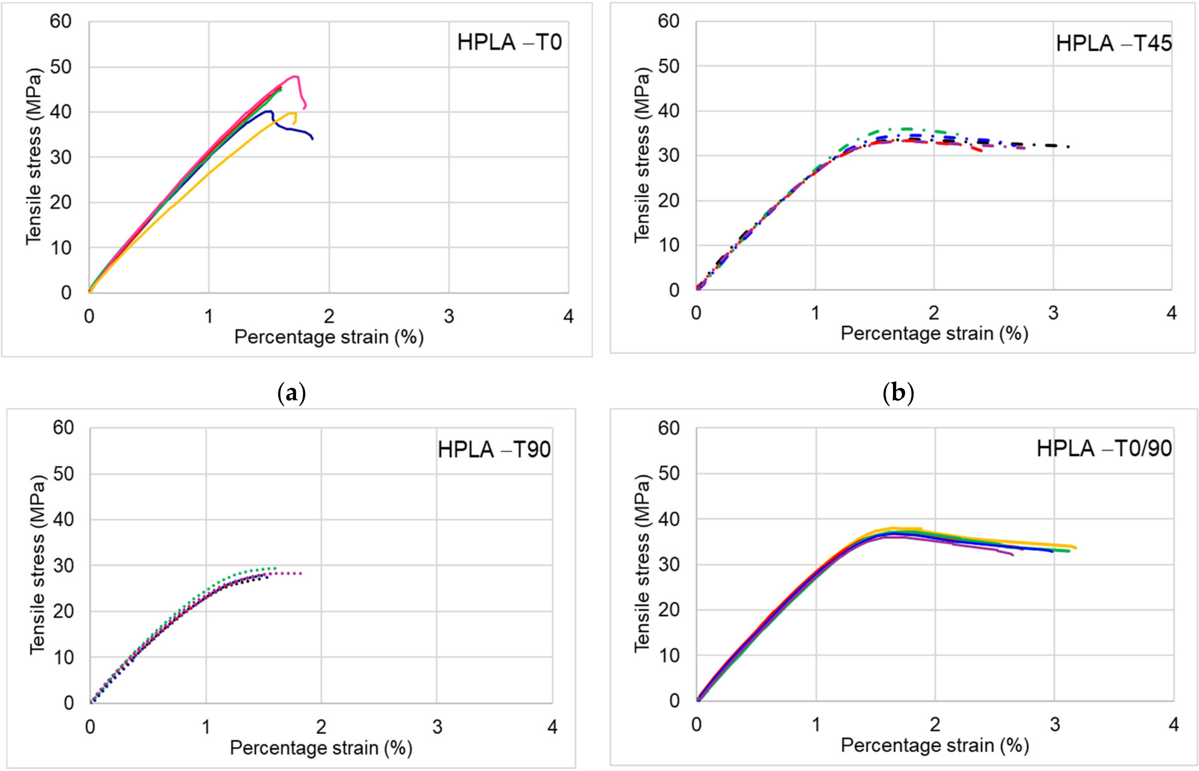

(d)

Figure S2. Characteristic curves of HPLA samples with different raster orientation subjected to tensile: (**a**) stress-strain curves for HPLA–T0; (**b**) stress-strain curves for HPLA–T45; (**c**) stress-strain curves for HPLA–T90; (**d**) stress-strain curves for HPLA–T0/90.

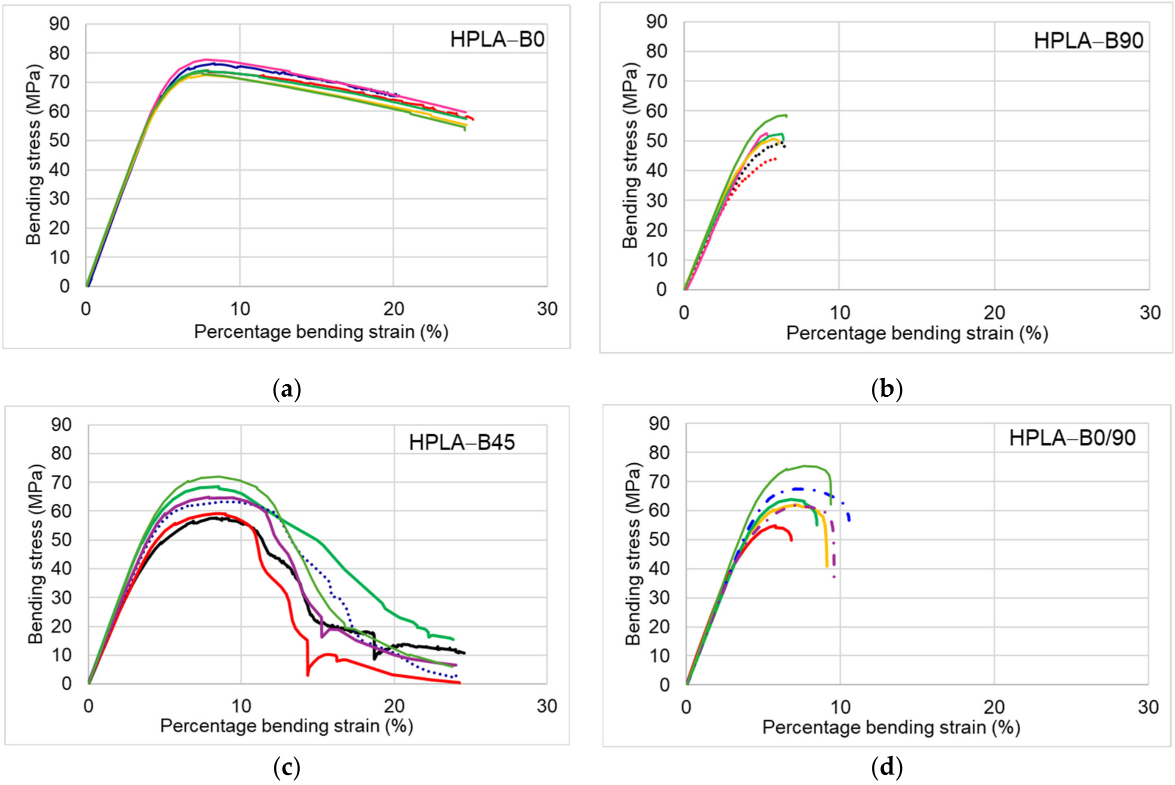

Figure S3. Characteristic curves of HPLA samples with different raster orientation, subjected to bending: (**a**) stress-strain curves for HPLA–B0; (**b**) stress-strain curves for HPLA–B90; (**c**) stress-strain curves for HPLA–B45; (**d**) stress-strain curves for HPLA–B0/90.
